# Effects of the presence of a cell phone and exposure to natural environments on remote associates task performance

**DOI:** 10.1038/s41598-022-13634-y

**Published:** 2022-06-09

**Authors:** Wenjuan Liu, Akihiko Dempo, Tsukasa Kimura, Tomoya Kawashima, Kazumitsu Shinohara

**Affiliations:** 1 Graduate School of Human Sciences, Osaka University, 1-2, Yamadaoka, Suita, Osaka 565-0871 Japan; 2 Faculty of Science and Technology, Seikei University, Musashino, Tokyo Japan; 3 The Institute of Scientific and Industrial Research, Osaka University, Suita, Osaka Japan

**Keywords:** Psychology, Human behaviour

## Abstract

In today’s advanced information society, creativity in work is highly valued, and there is growing interest in the kinds of work environments that produce more creative outcomes. Recent researchers have demonstrated that when environmental factors change a worker’s attentional state to a diffused state, the worker has access to more information than usual, which can contribute to creativity. Here, we examined whether manipulating environmental factors (the presence of a cell phone and exposure to natural environment) that could affect such attention states would improve performance on the Remote Associates Task, a measure of creativity. Our results showed that the presence of a cell phone increased creative performance regardless of immersion in natural environment. In contrast, exposure to nature did not facilitate creative performance; instead, feelings of pleasure increased, and frustration decreased. These results suggest that the presence of a cell phone can enhance creativity by influencing workers’ attentional states. The current study provides a meaningful approach to enhancing creativity by modulating attentional states through environmental factors. It also highlights the essential features of environmental factors that can moderate creative abilities.

## Introduction

In today’s advanced information society, it is now both possible and desirable to acquire information anytime and anywhere. Because mobile devices such as cell phones are, by definition, portable, users can obtain and exchange information whenever they choose as long as internet access is available; phones have also become the most common way to connect with others, primarily in the form of posts and exchanges on social network platforms. Meanwhile, cell phones are used for not only personal but also work-related purposes^1,2^ because of their great convenience. Cell phones today are considered essential for communication and not kept aside during both work and non-work hours. In addition, with the development of internet communication technology, work styles that are not restricted by location are being realized, and intellectual workers are becoming engaged in work in a variety of environments^3^. This change in work styles has increased the importance of cell phones at work.

In an advanced information society, simple and mechanical tasks will be automated, while human workers will be required to engage in tasks that require a high level of creativity^4–8^. For this reason, there has been a great deal of interest in enhancing the creativity of workers in various environments. The literature on creative research traditionally distinguished creative thinking into two types: divergent and convergent^9^. The first refers to creative cognitive processes of generating an original and novel idea (i.e., Alternative Uses Task^9^), whereas the second refers to those of associating different ideas to determine a single, correct solution to a problem (i.e., the Remote Associates Test [RAT]^10^). In this context, some researchers have suggested that adding situational factors can enhance creativity although creative tasks require a different creative thinking. For instance, physical disorderliness (e.g., how worse things are stored in the environment) is one environmental characteristic that enhances creative behaviors. Vohs et al.^11^ reported that participants in an environment with high-level disorderliness performed better in the Alternative Uses Task^9^ (e.g., asking participants to think of as many uses as possible for simple objects, such as bricks, shoes, or paper clips), which assess the creative divergent thinking process. In addition, Zhu et al.^12^ found that participants in moderate noise (70 dB) were able to perform a task on creative convergent thinking (the RAT^10^) better than those in high (85 dB) and low (50 dB) noise. With this noise study, the researchers also explored the underlying mechanism of the impact of noise on creativity; their findings suggested that moderate levels of everyday background noise can induce processing disfluency and activate an abstract style of cognitive processing that increases creativity. It is implied that manipulating attention through situational factors can drive optimal performance of convergent creative tasks in the workplace. Based on the theoretical framework of the relationship between creativity and attentional function (i.e., nonfocused attention), we aimed to explore possible ways to improve convergent creativity through situational factors in the present study.

People in a nonfocused attentional state are more creative than those in a focused state of attention^13–17^. Related studies^18,19^ have found that those who performed better on a creative task^20,21^ (e.g., Wallach–Kogan creativity tasks and Torrance Tests of Creative Thinking) showed poorer performance on cognitive tasks (e.g., divided attention task and the Stroop task). This is because people who have better attention control suppress task-unrelated information and maintain their focus on task-related information^22^; in those findings, the creative people were more likely to notice information that was not relevant to the task and thus to be distracted. In support of this conclusion, in other studies, lower ability to inhibit the influence of task-irrelevant stimuli was associated with higher creative performance^23,24^. Moreover, researchers have found that highly creative participants are not only more likely to be distracted by irrelevant information but also able to derive potential cues from that information^15^. This suggests that acting creatively lies in being able to perceive and utilize more task-unrelated information by loosening the attentional filter, which passes only task-related information. This attentional state is referred to as unfocused attention, nonfocused attentional state, diffuse attention, and leaky attention^13^.

Unfocused attention is not the result of personal characteristics of highly creative people but is observable in general populations when external factors facilitate the unfocused state. One of the factors that cause nonfocused attentional states is distraction, in which attention is drawn to an object unrelated to the task. For instance, in contrast with young adults, older adults who are vulnerable to distraction^25^ can use “distracting” information to obtain cues that are actually relevant to solutions^26^. Similarly, Jarosz et al.^27^ found that intoxicated individuals (i.e., blood alcohol content of approximately 0.075) generated more solutions to the RAT^10^ in less time than did nonintoxicated individuals. These results indicate a direct relationship between unfocused attention and creativity and suggest that inducing unfocused attention with external factors can foster creativity.

In the workplace, cell phones may trigger nonfocused attentional states, which in turn can foster creativity. In one recent study, the presence of cell phones caused cognitive failures^28^, and in other studies, the presence of a cell phone induced poor performance on cognitive tasks by reducing attentional resources available for the task^29,30^. Such effects can be attributed to attentional capture by external stimuli^29^, unconscious task-irrelevant thoughts^31,32^, and/or being in the state of permanent readiness to respond to one’s smartphone^33^. That is, the presence of cell phones can moderate the distribution of attention, reducing the amount of attentional resources directed to the task while slightly increasing the amount of attentional resources allocated to task-irrelevant stimuli. In other words, the presence of a cell phone can make users more sensitive to stimuli and information that is not task-related—that is, more easily distracted. In real-world circumstances, a person with a cell phone nearby might be in a nonfocused attentional state; therefore, we theorized that the presence of a cell phone could shift employees into unfocused attention.

Another way to create a state of nonfocused attention is through contact with nature. In natural environments, the variety of natural stimuli (e.g., waterfalls, birdsong, wind, and green trees) can involuntarily attract our attention^34^, and this attention capture is effortless; findings indicate that immersion in nature can modulate attention. Researchers found higher rates of gaze shifting in natural environments than in urban ones^35^, and other researchers found that after exposure to nature, autonomic arousal (heart rate) and the efficiency of spatial attention both decreased^36^. Accordingly, the attentional state in an environment with a variety of natural stimuli is more likely to be dispersed and closer to nonfocused than will be the case in environments without stimuli (e.g., an office). Therefore, we expect that exposure to nature will have the similar effect of distracting attention from the main task as the effect of the presence of a cell phone.

Indeed, several studies have shown that exposure to nature increased creativity^37–41^. Participants can benefit from experiencing exposure to nature prior to subsequent creative tasks^37,38,42^ and immersing in an environment with natural elements during tasks^39,43,44^. Meanwhile, wild (real nature) and indoor (pseudo-nature with natural elements) natural environments can induce the effect of exposure to nature, although the effect in an indoor environment was only evident among female participants^39^ and is unlikely to induce as large an effect as that from wild natural environments. Despite these findings, the underlying mechanism of exposure to nature regarding creativity lack clarity, especially in simulated natural indoor environments. The effects of exposure to nature on cognitive activity are generally interpreted in terms of Attention Restoration Theory^34^ (a variety of natural stimuli attract involuntary attention, thus restoring voluntary attentional resources) and Stress Reduction Theory^45,46^ (directing attention to vigorous, green natural stimuli induces positive emotions and low stress). However, other studies have reported that exposure to nature can also facilitate performance in typical cognitive tasks that assess lower-order attentional functions, such as working memory and selective attention^47^, claiming that this facilitation should be due to the recovery of attentional resources through exposure to nature. Although these theories can explain the effects of exposure to nature in certain cases, they cannot account for the findings that such improvements in attentional functioning frequently exert negative effects on creativity^48^. Thus, it is more likely that the effects of exposure to nature on creativity that depend on distractive (i.e., nonfocused) attentional states can be achieved by mildly directing attention to a variety of stimuli rather than restoring attention. If so, the presence of natural elements is required during creative tasks but not before or during breaks. Furthermore, related studies^44,49^ used head-mounted displays to induce an immersive environment that enables virtual contact with nature. However, using such specialized devices in actual situations is typically and practically difficult. Thus, examining the extent to which non-immersive contact with nature enhances convergent creativity without using special devices would be of practical significance.

Taken together, we had two goals with the current study: examining whether the presence of a cell phone would enhance creativity and examining the relationship between creativity and exposure to nature, which is known to affect creativity. Accordingly, we hypothesized that exposure to nature during a convergent creativity task would regulate the attentional state of the participants to be nonfocused, which improves creative abilities.

## Method

### Participants

The participants were 32 adults (16 men, 16 women) recruited by a participant-recruiting agency (aged 18–31 years; *M*_*age*_ = 21.34, *SD*_*age*_ = 2.42). A minimum sample size of 32 was estimated via a priori power analysis^50^ using G*Power 3.1.9.4 (with settings of power = 0.80, alpha = 0.05, and effect size *f* = 0.4) based on a previous report^37^ (Cohen’s d = 0.86). Twenty-seven were right-handed, four were left-handed, and one was ambidextrous. This study was approved by the Behavioral Research Ethics Committee of the School of Human Sciences, Osaka University in Japan (HB019-080). Written informed consent was obtained from all participants. All methods were performed in accordance with the relevant guidelines and regulations.

### Experimental apparatus and manipulations (Fig. 1)

**Figure 1:**
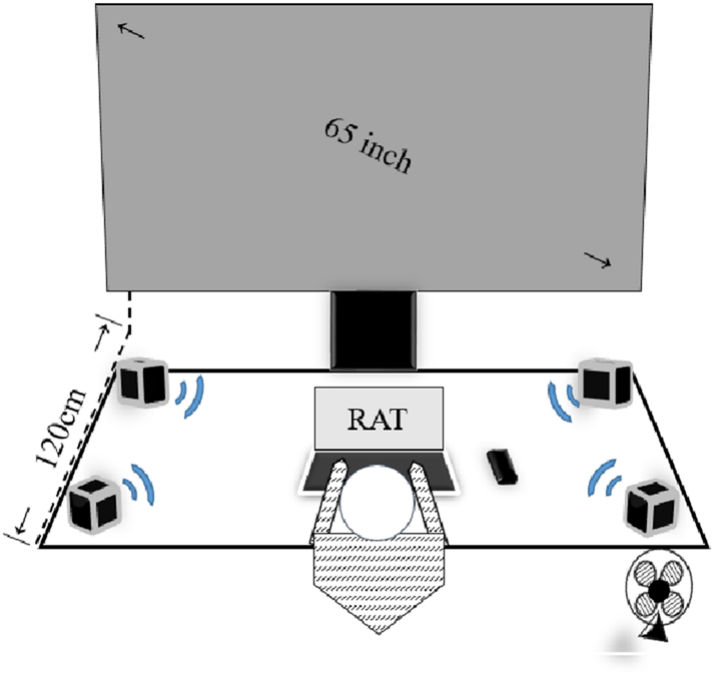
The experimental environment.

A video player (Panasonic, DP-UB9000) presented videos of natural scenes provided by LandSkip Inc. (forest, waterfall, coast; https://www.landskip.jp/) on a 65-inch LED monitor (JAPANNEXT, JN-V6500UHDR) located 120 cm from the participant’s seat. A stereo system (Pioneer, 5.1ch HTP-S363) with surround sound speakers (Pioneer S-H100) was used to project the videos’ stereoscopic sound at a volume of 70 dB. A breeze was created by a small fan placed on the side of the desk. The computer (Mouse, MB-B505S-M2S1) was set in the center of the desk (120 cm [W] × 60 cm [H]), and the software PsychoPy (ver. 1.83.04, Peirce, 2007, 2009) presented the task stimulus. A smartphone (Apple iPhone 11) was placed on the side of participants’ dominant hand.

Skin conductance level (SCL) represents the tonic level of a skin response and reflects general changes in arousal^51^. Skin conductance was recorded using an electrodermal activity amplifier MaP1720CA and unit AP-U030 (Nihonsanteku) with two circular electrodes (1-cm diameter, Mets Inc.); these were attached to the medial phalanx surfaces of the middle and index fingers of the nondominant hand. These data were recorded using input monitor software (Nihonsanteku), and the sampling rate was 500 Hz with a 0–15 Hz digital band-pass filter applied.

### Search task

The search task was a dummy task and conducted before the RAT to make participants aware of the presence of the cell phone. Participants were given a list of 10 Chinese characters and asked to use the designate phone to find 10 common two-word phrases (e.g., “内庭”: inside court) that contained the given characters (e.g., “内”: inside); they had 5 min to collect phrases and write them in the answer column.

### RAT

Creativity was measured by the Japanese version of the RAT^52^—used in a previous study^37^ as a convergent creative task. The RAT requires participants to find a single associated word that can be combined with each of the given three words (either being placed before or after it) to make a common word or phrase. For example, a RAT item including three words (e.g., dream, break, and light) can be solved by the associated word (i.e., “day”). The Japanese version of RAT has the similar rules as the English version in that participants were presented with three Chinese characters (2.5° × 2° visual angle) on the center of a screen and asked to write down a common Chinese character associated with each presented character.

### Assessing affect and mental workload

To investigate the affective impacts of the presence of a smartphone and of exposure to nature, we used the Positive and Negative Affect Schedule^53,54^ (PANAS) to assess the changes in affect between before and after participants completed the RAT with or without the phone nearby. For the current study, a native Japanese speaker translated the PANAS, and the internal reliability was high: Cronbach’s alpha = 0.847 and 0.844 for positive and negative affect, respectively.

To determine what internal processes are activated by exposure to nature to improve performance on a creativity task, it is necessary to measure affect^55^ (valence and arousal), physiological response to stress, and mental load. Here, we measured mental load^56^ with five items from the NASA TLX^57^ (desire for knowledge, own performance, frustration, effort, and overall workload) as a subjective assessment and measured stress based on physiological response, specifically skin conductance.

### Experimental flow and procedure

**Figure 2:**
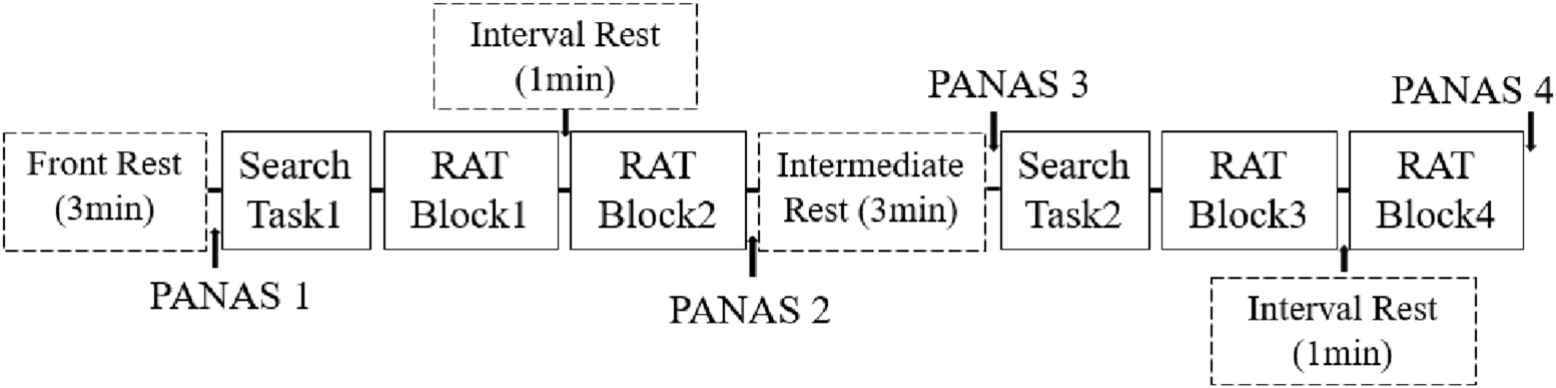
The sequence of single day’s experiment.

Participants took part in experiments over two days, one in the presence of a smartphone and the other in absence for counterbalance. Only participants in the exposure to nature condition completed the two-day experiment in an environment with natural elements; those in the control condition completed the two days experiment in an environment without natural elements. Based on a preliminary experiment, we set the RAT items in each phone condition at identical, moderate difficulty (i.e., average correct rate of each task set: 60%). In addition, the order of the RAT items in each phone condition was all randomized. The procedures for both days’ experiments were identical except that the participants were required to complete a practice task on the first day. The experimental flow for one day is shown in Fig. 2.

After participants finished the practice task in RAT, electrodes were pasted on the second joints of their forefingers and middle fingers on their nondominant hands, and then the SCL recording began. Before the participants entered the search task, the SCL recording lasting 3 min (i.e., front rest) were used as the baseline. Next was the main task session: in the nature-present condition, a video of natural scenes and related settings were presented (see the "Experimental apparatus and manipulations" section). These natural elements were present until the completion of the experiment. The main experiment had two sets of tasks; one task set included a search task and two blocks of the RAT, with 3 min of rest between the blocks. In both phone conditions, the smartphone was initially placed in an identical location before the phone search task and was switched on throughout the experiment. In the phone-absent condition, the experimenter took away the phone *after* the search task and put a mobile battery on the table that was similar in size and weight to the phone; this time, participants could see and were able to touch the battery but not use it.

In the two blocks of the RATs (9 trials per block), participants were required to complete them successively and had a 1-min rest interval between the blocks. In one trial, there is a RAT thinking section with a 45-s limit, followed by a RAT answering section with no time limit. In the thinking section, the characters were presented for 45 s, and the participants were asked to think of a correct answer within that time. In the answering section, the instructions for the answer method and the current trial number were presented instead of the characters. If the participants could find the answer within 45 s, they were asked to press the space key to enter the answering section and then write their answers on the designated response sheet. If the participants failed to find the correct answer in 45 s, the screen automatically switched to the answering section. The participants were instructed not to continue thinking about the RAT after the screen switched.

The participants completed the PANAS^54^ four times: before the first RAT block, after the second, before the third, and after the fourth. Participants also answered questions about mental load (5 items from NASA-TLX) and about their feelings toward natural environments (9-point scale measuring valence and arousal) after the experiment. On the second day, participants completed the Problematic Use of Mobile Phone (PUMP) questionnaire^58^ (translated by a native Japanese speaker) and the question of demographic information. The PUMP is a scale that measures the level of dependence on mobile phones and was used to control and exclude the influence of between-group differences in addictive behavior.

### Experiment design and hypothesis

This experiment followed a 2 (exposure to nature: absent or present, varied between participants) × 2 (phone presence: present or absent, varied within participants) factorial design. The hypothesis of this study is as follows:

- **H1**: (a) Participants with a smartphone nearby will perform the creative task better than participants who do not have a smartphone nearby (b) The score of negative affect will increase in the presence of the smartphone.
- **H2**: Participants will perform the creative task better in a nature-present condition than in a nature-absent condition.
- **H3**: Participants will experience (a) less mental workload, (b) stress, and (c) be more relaxed in a nature-present condition. This difference in subjective feelings will be reflected in (a) All or part of the items from NASA -TLX would decrease; (b) SCL will change less in a nature-present condition; and (c) The score of valence will increase and the score of arousal would decrease in a nature-present condition irrespective of cell phone presence.

In summary, performance of the RAT will be higher when a smartphone is present, nature elements are present, or both are present than when neither a phone nor nature elements are present. Additionally, we explored how these interactions would affect creativity performance.

## Results

### Data analysis

We discarded data from two participants because a program error was found for one participant and the other participant misunderstood an instruction for the RAT and could not follow the appropriate procedure. Thus, we analyzed the data of the remaining 30 participants.

To represent physiological arousal level changes, we calculated the averages of SCL values for front rest, intermediate rest, and blocks of the RATs^59^. Reactivity in response to every block was calculated by subtracting the average of baseline SCL values from the average of SCL values during the RAT blocks, which combined nature exposure (absence or presence) and phone (presence or absence) conditions; front rest as the baseline SCL corresponded to blocks 1 and 2, and intermediate rest corresponded to the remaining blocks.

To confirm whether the individual differences in cell phone usage dependence affect the observed effect of exposure to nature on creativity, the PUMP score between the nature absence and presence conditions were analyzed for normality (Shapiro–Wilk normality test: mean_abs_ = 127.20, W_abs_ = 0.92, *p*_*abs*_ = 0.19; mean_pre_ = 116.13, W_pre_ = 0.97, *p*_*pre*_ = 0.89), equality of variances (F-test: *F*(1, 14) = 0.80, *p* = 0.69, 95% CI 0.27–2.40), and differences in means (T-test: *t*(28) =  − 0.93, *p* = 0.36, IC 95% =  − 35.41–13.28, *d* = 0.34), respectively. These results show that the two groups of PUMP scores followed a normal distribution with equal variance and no difference between the means was observed, indicating that any influence we observed between nature absence and presence could not be attributed to the individual inference of cell phone usage.

The angular transformed values of the ratio of correct RAT response and reaction time (key press reaction time in the thinking section of the correct answer) were analyzed using two-way analysis of variance (ANOVA) with phone presence and exposure to nature as factors. We analyzed SCL using a three-way ANOVA with phone presence, exposure to nature, and RAT block as factors. The score of negative and positive affect was analyzed by a three-way ANOVA with measurement timing (marked as P1, P2, P3, and P4), phone presence, and exposure to nature as factors. Subjective ratings other than PANAS were analyzed using a two-way ANOVA as in the analysis of hit rate. All degrees of freedom were adjusted using Chi-Muller’s epsilon. All the multiple comparisons used Shaffer’s multiple-comparison procedure. The details of the nonsignificant results are available at http://osf.io/x93j6/.

### RAT performance

**Figure 3:**
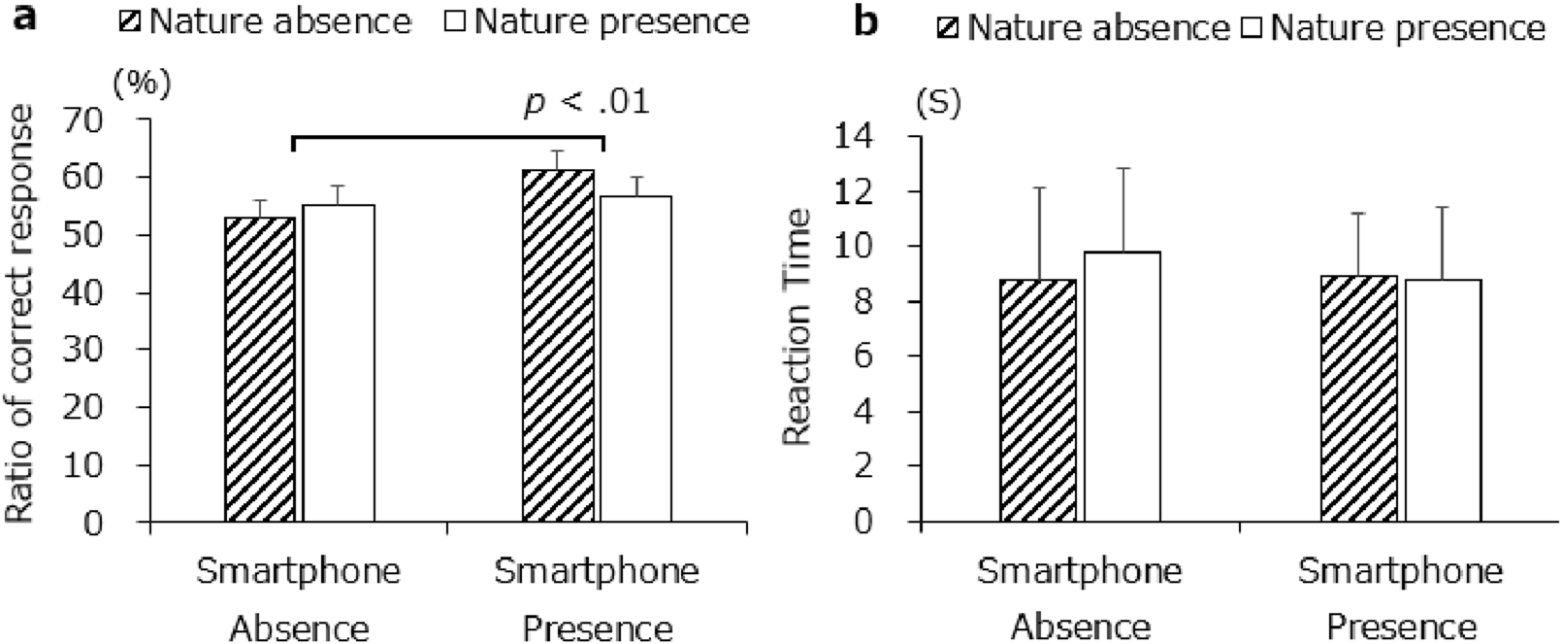
Ratios of (**a**). correct RAT response and (**b**). reaction time. Error bars depict standard error values.

Figure 3 shows the ratios of correct responses (left); the main effect of phone presence was significant, *F*(1, 28) = 11.06, *p* < 0.01, *η*^2^ = 0.0625, whereas the main effect of exposure to nature was not significant, *F*(1, 28) = 0.30, *p* = 0.59, *η*^2^ = 0.0083. The two-way interaction for nature and phone presence was also not significant, *F*(1, 28) = 1.46, *p* = 0.24, *η*^2^ = 0.0083. Figure 3 (right panel) shows that we observed no significant difference in either main effects or interactions in the results for reaction time. Irrespective of nature condition, the participants with a smartphone nearby generated more correct responses than did those who had only a mobile battery present. In short, the presence of a smartphone increased creativity task performance, supporting H1a.

### SCL change

**Figure 4:**
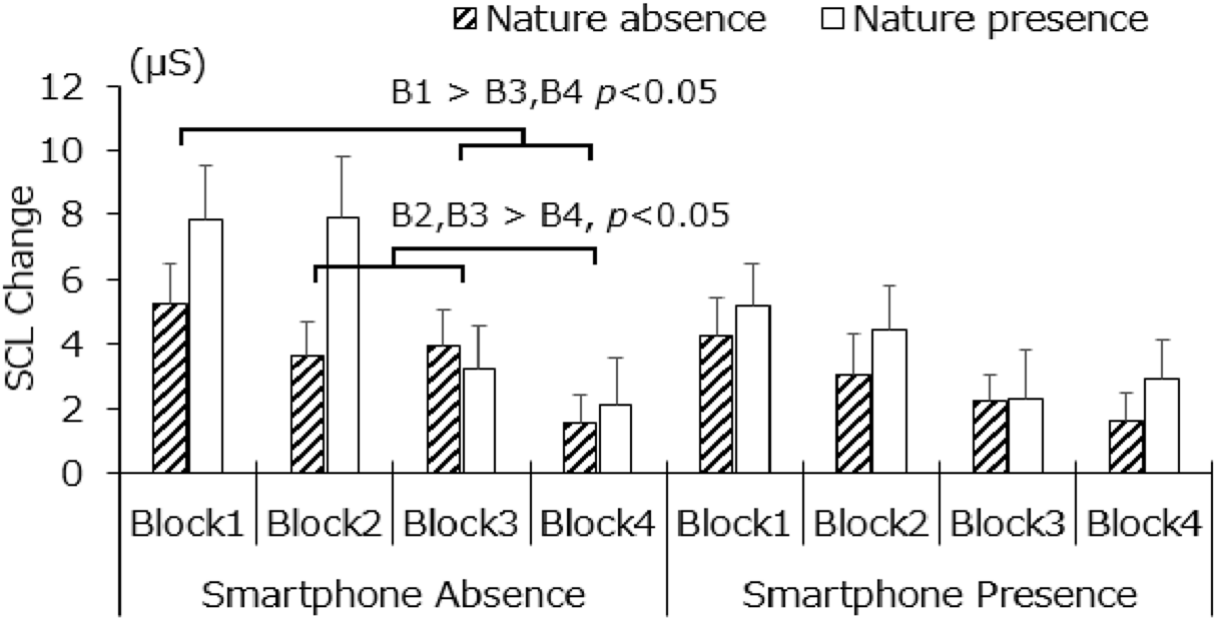
SCL changes. Error bars depict standard error values.

The main effect of the block was significant, *F*(1.35, 37.85) = 9.24, *p* < 0.01, *η*^*2*^ = 0.0751, whereas the main effects of phone presence, *F*(1, 28) = 2.38, *p* = 0.13, *η*^2^ = 0.0129, and exposure to nature, *F*(1, 28) = 1.05, *p* = 0.31, *η*^2^ = 0.0156, were not. The two-way interaction for the block and phone presence was significant, *F*(2.24, 62.6) = 3.26, *p* < 0.05, *η*^*2*^ = 0.0086, and the simple main effect of block was significant in the phone-absent condition, *F*(1.66, 46.61) = 12.54, *p* < 0.01, *η*^2^ = 0.1092; this latter finding indicated that SCL changed more in block 1 (6.53 μS) than in blocks 3 (3.57 μS) and 4 (2.26 μS; *p*s < 0.05); SCL in blocks 2 and 3 was also higher than that in block 4 (*p*s < 0.05). Whereas the simple main effect of the block was marginally significant in the phone-present condition, *F*(1.48, 41.42) = 3.53, *p* = 0.05, *η*^2^ = 0.0498, there were no significant differences between the blocks. The simple main effect of phone presence was only significant in block 2, *F*(1, 28) = 5.89, *p* < 0.05, *η*^2^ = 0.0334, which indicated that SCL changed more in the absence of a smartphone than in its presence in that block (3.56 μS > 1.81 μS). In the phone-absence condition, the SCL changes decreased over time, but we did not observe this tendency when the phone was present. Meanwhile, there was no significant difference between nature conditions; thus, H3b was not supported. Figure 4 graphically presents the SCL changes.

### Subjective affect ratings

**Figure 5:**
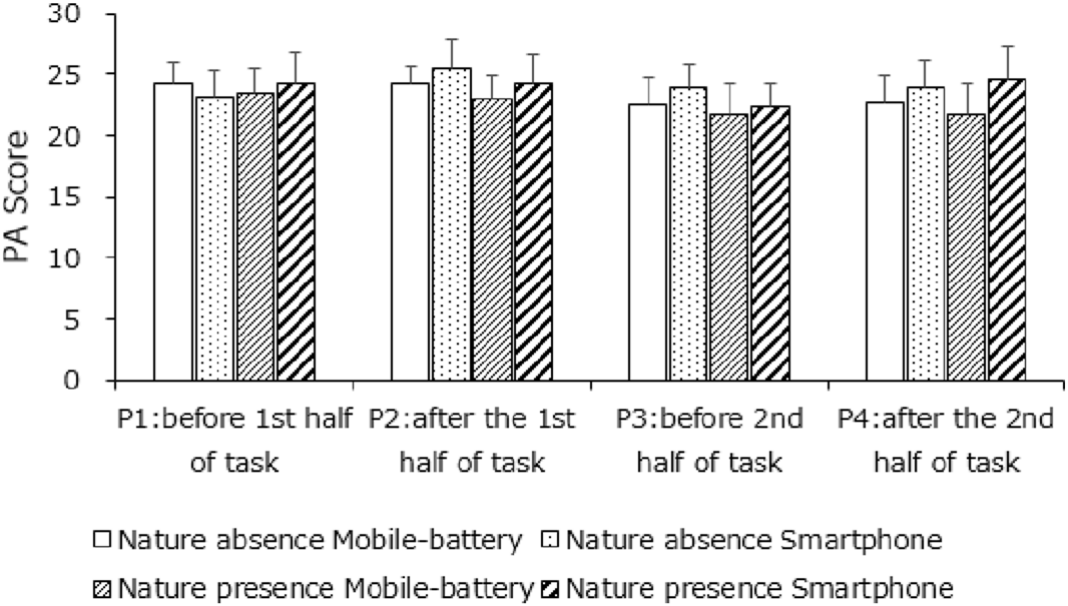
Mean positive affect scores. Error bars depict standard error values.

**Figure 6:**
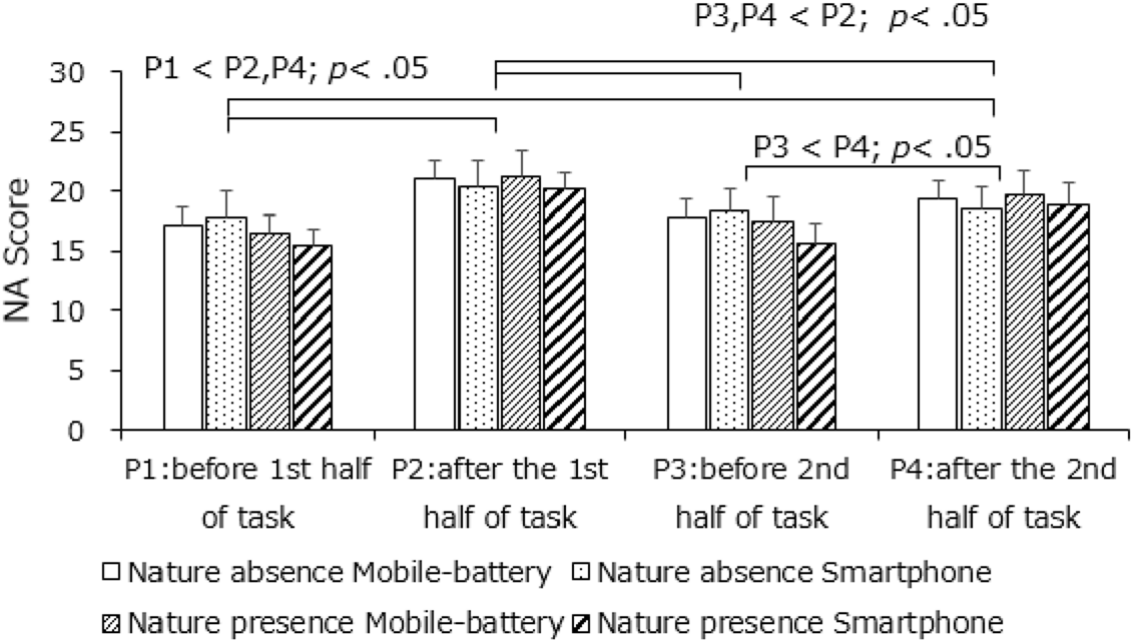
Mean negative affect scores (lower). Error bars depict standard error values.

Figures 5 and 6 presents the PANAS results for affect ratings in the presence or absence of a nature environment. No main effects or interactions were significant for positive affect, whereas for negative affect (one scale of the PANAS), the main effect of measurement timing was significant, *F*(2.58, 72.12) = 10.05, *p* < 0.001, *η*^2^ = 0.0523; neither the other main effects nor the interactions were significant. Participants showed higher scores for negative affect after completing the first half of the tasks (P1 < P2, P4; P3 < P4, *p*s < 0.05) that decreased after rest (P2 > P3, *p* < 0.05); moreover, the negative affect increment in the first half of the tasks was larger than that in the second half (P1 = P3, P2 > P4, *p* < 0.05). These results did not reflect any difference due to the presence of a smartphone; thus, H1b was not supported.

**Figure 7:**
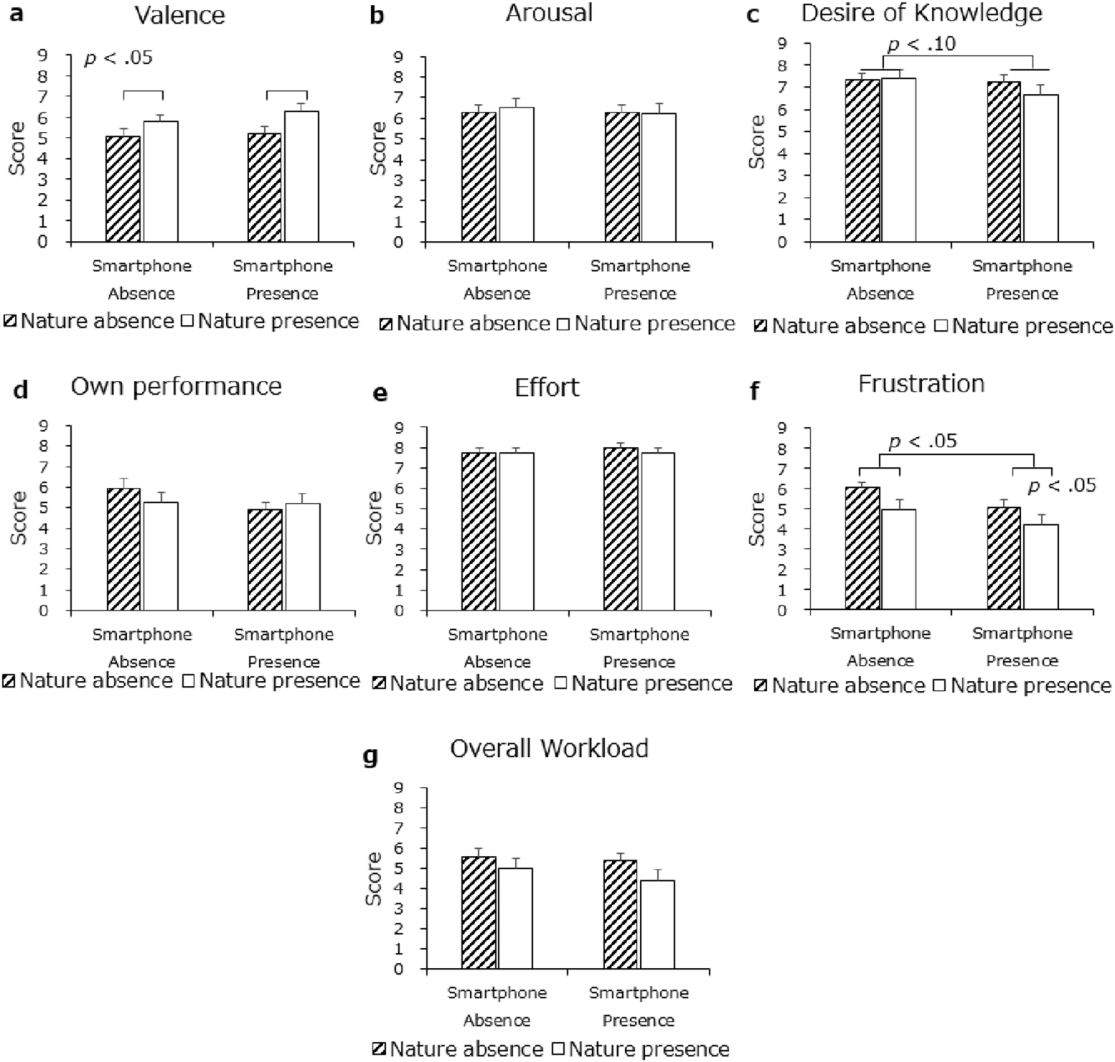
Mean subjective evaluations: (**a**) valence, (**b**) arousal, (**c**) desire of knowledge, (**d**) own performance, (**e**) effort, (**f**) frustration, and (**g**) overall workload. Error bars depict standard error values.

Figure 7 displays the remaining subjective ratings for all participants. For valence, the main effect of exposure to nature was significant, *F*(1, 28) = 5.40, *p* < 0.05, *η*^2^ = 0.0903, but not that for phone presence, *F*(1, 28) = 0.65, *p* = 0.43, *η*^2^ = 0.0100, which reflected that participants felt more pleasure from the presence of nature than they did in its absence irrespective of phone presence conditions. No two-ways interaction was detected, *F*(1, 28) = 0.20, *p* = 0.66, *η*^2^ = 0.0031. For arousal, neither main effect nor interaction was significant. Therefore, H3c was partially supported.

For desire of knowledge, the main effect of phone presence was marginally significant, *F*(1, 28) = 3.38, *p* = 0.07, *η*^*2*^ = 0.0244, whereas the main effect of exposure to nature was not, *F* (1,28) = 0.26, *p* = 0.61, *η*^*2*^ = 0.0071. No two-ways interaction was detected, *F*(1, 28) = 1.62, *p* = 0.21, *η*^*2*^ = 0.0117. The participants’ desire for knowledge was more conscious when the phone was absent than when it was present, which indicated that the mental demand without a smartphone was higher, and the participants had to attempt to retrieve more knowledge to arrive at a correct answer.

For frustration, both the main effect of exposure to nature, *F*(1, 28) = 4.38, *p* < 0.05, *η*^2^ = 0.0782, and the main effect of phone presence, *F*(1, 28) = 4.54, *p* < 0.05, *η*^2^ = 0.0588, were noted. No two-ways interaction was detected, *F*(1, 28) = 0.10, *p* = 0.75, *η*^2^ = 0.0014. Participants showed more frustration in the absence of nature than in its presence and more frustration in the absence of a smartphone than in its presence. For the remaining subjective ratings, all main effects and interactions were nonsignificant. Figure 7 presents all the mean subjective ratings. In the overall rating of five items from the NASA task load index, mental load in the form of frustration was lower in the nature environments, which partially supported H3a.

## Discussion

The main aim of the current study was to determine whether the presence of a cell phone and/or exposure to nature would affect creativity. In this study, participants with a smartphone nearby performed better on RATs (Fig. 3a) and reported less frustration and less desire for knowledge than did participants with only a mobile battery nearby (Fig. 7c, f); that is, the mere presence of a cell phone fostered creativity. Regarding the effects of exposure to nature, participants reported more pleasure and less frustration in its presence than in its absence, although we did not observe related nature benefits in creative performance.

The first and most vital finding in the current study is that a cell phone presence after using it facilitated the performance of the sequent creative task (i.e., RAT: Fig. 3a), indicating that the presence of a cell phone can increase creative outcomes. This result could be induced by adjusting the participants’ attentional state to a nonfocused one. A cell phone can be used as an extended technological device to support offloading cognitive demand^60,61^ (e.g., excessive information processing). When people engage in a task utilizing a cell phone, their attention may be diffused to plural information sources rather than converging to a single source because diffused attentional state has many advantages (e.g., increased efficiency of information processing). In the current case, it is plausible that the state of diffused attention that was induced by using a cell phone was maintained during the subsequent creative task in the phone-present condition. Therefore, it is possible that the presence of a cell phone distracted the participants’ attention but simultaneously led to unfocused attention that enabled them to access more information to perform better in the creativity task. Notably, a previous study^62^ that used a luminance detection task to directly observe distraction by a cell phone, which was presented through a similar method as that of this study. That is, although the current study did not measure the available attentional resources during the creative task directly, it can be assumed that distraction is also caused by the cell phone herein.

Further, it was found that participants reported a lower subjective mental load (reflected in frustration and desire for knowledge) as they completed the RATs in the presence of a cell phone (Fig. 7c, f), suggesting that they were aware to some extent that the presence of a cell phone facilitated their cognition. Moreover, we found that the SCL changes decreased gradually as the task progressed but only in the absence of a cell phone (Fig. 4). This finding suggests that the presence or absence of a cell phone has some effect on arousal, but the underlying mechanism is currently difficult to interpret. Future work is necessary to further examine the relationship between the presence of a cell phone and corresponding changes in physiological arousal level.

In this study, it is not clear whether the facilitative effect of the presence of cell phones on creativity is stronger than the disruptive effect produced by distraction. Although multiple researchers have observed negative effects of cell phone presence^28–32,62,63^, many others have concluded that it is difficult to replicate the effects of a cell phone’s presence and that the effects can even be described as weak^64–67^. It is likely that other potential factors (e.g., task meaningfulness to participants and individual differences in emotion-related impulsivity) can moderate or limit the negative effects such that they are not evident in a wide range of situations. The next important matter is determining the relative strength of the creativity-fostering effects of the presence of cell phones compared with their disruptive effects on attention, and it is also necessary to consider how to increase the facilitative effects while limiting distraction.

Inconsistent with related studies on the benefits of exposure to nature for creative outcomes^37–40^, we found no significant influence of natural environments on creative performance, while we only observed the moderated effect on the affective aspect. The positive mood changes are reflected by the self-rating of valence and frustration, so participants in a natural environment felt more pleasant and could reduce the negative affective consequences that may accompany performing a task (i.e., RAT) with a relatively high cognitive load. Increased positive mood as one of the typical affective benefits of exposure to real natural environment^68^ (e.g., forests and park) was observed in the current indoor natural setting (Fig. 7a, f), implying that the simulated indoor natural environment has the capability to elicit similar affective benefits as the real natural environment. However, the expected improvements in creative performance through exposure to nature, as in Atchley’s^37^ study, were not evident. One possible explanation for this discrepancy involves the time constraint employed herein. Atchley^37^ did not place limits on respondents’ time to answer; we imposed a 45-s time limit, instructing participants to follow the given instructions in producing their responses. Only responses that were made within this time limit were considered correct. Creative thinking is regarded as a high-order cognitive process not driven by speed of information processing^69^; hence, unlimited response duration can provide an opportunity for individuals to maximize their creative potential in the performance of tasks measuring creativity. That is, the observed facilitation effect of exposure to nature under the condition of no time limit might be larger, compared with a time limit situation such as that of the current study. Thus, we speculate that this time limitation may have weakened the effect of exposure to nature observed at the behavioral level. Future research is needed to consider time in addressing the effects of an indoor environment with nature elements on creativity.

The current study has several limitations. First, we used a designated cell phone rather than each participant’s own cell phone to strictly control factors such as phone brand, series, and color. However, the impact of their own phones should be more realistic and richer, given the complexity of the intrinsic causes of cell phone presence effects. Second, although we believe that the presence of a cell phone affects creativity by altering one’s attentional state to allow processing of more task-irrelevant information, we did not measure the ability of participants to generate information that is slightly less relevant to task such as remote associated words, which were measured in a related study^70^. In other words, the present results alone do not rule out the possibility that cell phones are fostering creativity in other ways (e.g., affecting motivation). Therefore, we can only say that the current findings provide indirect evidence, and further research is needed to directly confirm the underlying mechanism of the impacts of a cell phone’s presence on creativity. Third, although the current study assumed that improved creative ability is associated with nonfocused attentional state, the most effective method for verifying this assumption is to test whether creative performance could be enhanced when the participants performed a monitoring task that assesses variation in attentional state during a creative task. Thus, further studies using this method are necessary. Fourth, our study used a non-immersion natural contact method, which may differ from the well-known traditional method. Therefore, the fact that the types of exposure to nature may lead to the lack of effects regarding exposure to nature cannot be ruled out. Future studies should consider other types of exposure. 

The present study provides experimental evidence that the presence of a cell phone can positively affect creativity. Because this influence was tested in settings that replicate real-world work environments, our findings possibly reflect a more comprehensive expression of the impacts of a cell phone’s presence and will contribute to a better understanding of the impacts of cell phones on performance and creativity in work environments. In addition, our study adds to the body of knowledge on fostering creativity by demonstrating that so-called nonfocused attentional state might be crucial to linking situational and environmental factors to increased creative outcomes.

## Data Availability

Data and scripts are available on the Open Science Framework: http://osf.io/x93j6/.

## References

[1] Li L, Lin TTC (2018). Examining how dependence on smartphones at work relates to Chinese employees’ workplace social capital, job performance, and smartphone addiction. Inf. Dev..

[2] Neștian ȘA, Tiță SM, Turnea E-S (2020). Using mobile phones at work in personal and professional information processes. Sustainability.

[3] Byström, K., Ruthven, I. & Heinström, J. Work and information: which workplace models still work in modern digital workplaces? *Inf. Res.* **22**, CoLIS Paper 1651 (2017).

[4] Zhou J, Hoever IJ (2014). Research on workplace creativity: a review and redirection. Annu. Rev. Organ. Psychol. Organ. Behav..

[5] Corazza GE (2017). Organic creativity for well-being in the post-information society. Eur. S J. Psychol..

[6] Glăveanu, V. P., Ness, I. J. & de Saint Laurent, C. Creativity, learning and technology: opportunities, challenges and new horizons. *Creativity Res. J.* **32**, 1–3 (2020).

[7] Amabile TM (1983). The social psychology of creativity: a componential conceptualization. J. Pers. Soc. Psychol..

[8] Dul J, Ceylan C, Jaspers F (2011). Knowledge worker creativity and the role of the physical work environment. Hum. Resour. Manag..

[9] Guilford, J. P. *The nature of human intelligence* (McGraw-Hill, 1967).

[10] Mednick SA (1968). The remote associates test. J. Creat. Behav..

[11] Vohs KD, Redden JP, Rahinel R (2013). Physical order produces healthy choices, generosity, and conventionality, whereas disorder produces creativity. Psychol. Sci..

[12] Mehta R, Zhu RJ, Cheema A (2012). Is noise always bad? Exploring the effects of ambient noise on creative cognition. J. Con. Res..

[13] Zabelina, D. Attention and creativity in *The Cambridge handbook of the neuroscience of creativity* (eds. Vartanian, O. & Jung, R.) 159–230 (Cambridge University Press, 2018).

[14] Wiley J, Jarosz AF (2012). Working memory capacity, attentional focus, and problem solving. Curr. Dir. Psychol. Sci..

[15] Ansburg PI, Hill K (2003). Creative and analytic thinkers differ in their use of attentional resources. Pers. Individ. Dif..

[16] Finke, R., Ward, T. B. & Smith, S. M. Reviews in *Creative cognition: theory, research and applications* 238–247 (MIT Press, 1992).

[17] Martindale, C. Creativity and connectionism in *The creative cognition approach* (eds. Smith, S. M., Ward, T. B. & Finke, R. A.) 249–268 (MIT Press, 1995).

[18] Rawlings D (1985). Psychoticism, creativity, and dichotic shadowing. Pers. Individ. Dif..

[19] Sharma S, Babu N (2017). Interplay between creativity, executive function and working memory in middle-aged and older adults. Creat. Res. J..

[20] Wallach M, Kogan N (1965). Modes of thinking in young children.

[21] Torrance, E. *The Torrance tests of creative thinking. Norms—technical manual. Research edition. Verbal tests, forms A and B. Figural tests, forms A and B* (Personnel Press, 1974).

[22] Miyake A, Friedman NP, Emerson MJ, Witzki AH, Howerter A (2000). The unity and diversity of executive functions and their contributions to complex frontal lobe tasks: a latent variable analysis. Cogn. Psychol..

[23] Carson SH, Peterson JB, Higgins DM (2003). Decreased latent inhibition is associated with increased creative achievement in high-functioning individuals. J. Pers. Soc. Psychol..

[24] Zmigrod S, Zmigrod L, Hommel B (2019). The relevance of the irrelevant: attentional distractor-response binding predicts performance in the Remote Associates Task. Psychol. Aesthet. Creativity Arts.

[25] May CP (1999). Synchrony effects in cognition: the costs and a benefit. Psychon. Bull. Rev..

[26] Kim S, Hasher L, Zacks RT (2007). Aging and a benefit of distractibility. Psychon. Bull. Rev..

[27] Jarosz AF, Colflesh GJ, Wiley J (2012). Uncorking the muse: alcohol intoxication facilitates creative problem solving. Conscious. Cognit..

[28] Cambier R, Van Laethem M, Vlerick P (2020). Private life telepressure and workplace cognitive failure among hospital nurses: the moderating role of mobile phone presence. J. Adv. Nurs..

[29] Ward AF, Duke K, Gneezy A, Bos MW (2017). Brain drain: the mere presence of one’s own smartphone reduces available cognitive capacity. J. Assoc. Consum. Res..

[30] Ito M, Kawahara JI (2017). Effect of the presence of a mobile phone during a spatial visual search. Jpn. Psychol. Res..

[31] Thornton B, Faires A, Robbins M, Rollins E (2014). Mere presence of cell phone may be distracting. Soc. Psychol..

[32] Stothart C, Mitchum A, Yehnert C (2015). The attentional cost of receiving a cell phone notification. J. Exp. Psychol. Hum. Percept. Perform..

[33] Vorderer P, Kohring M (2013). Permanently online: a challenge for media and communication research. Int. J. Commun..

[34] Kaplan S (1995). The restorative effects of nature: towards an integrative frame-work. J. Environ. Psychol..

[35] Stevenson MP, Dewhurst R, Schilhab TS, Bentsen P (2019). Cognitive restoration in children following exposure to nature: evidence from the Attention Network Task and mobile eye tracking. Front. Psychol..

[36] Laumann, K., G.arling, T., & Stormark, K. M. Selective attention and heart rate responses to natural and urban environments. *J. Environ. Psychol*. **23,** 125–134 (2003).

[37] Atchley RA, Strayer DL, Atchley P (2012). Creativity in the wild: improving creative reasoning through immersion in natural settings. PLoS ONE.

[38] Ferraro FM (2015). Enhancement of convergent creativity following a multiday wilderness experience. Ecopsychology.

[39] Shibata S, Suzuki N (2004). Effects of indoor plan on creative task performance and mood. Scand. J. Psychol..

[40] Alawad A (2012). Can we bring the natural environment into the art classroom? Can natural sound foster creativity?. Educ. Res. Rev..

[41] Plambech, T. & Konijnendijk van den Bosch, C. C. The impact of nature on creativity-a study among Danish creative professionals. *Urb. For. Urb. Green.* **14**, 255–263 (2015).

[42] Oppezzo M, Schwartz DL (2014). Give your ideas some legs: the positive effect of walking on creative thinking. J Exp. Psychol. Learn. Mem. Cognit..

[43] Chulvi V, Agost MJ, Felip F, Gual J (2020). Natural elements in the designer's work environment influence the creativity of their results. J Build. Eng..

[44] Fleury S, Blanchard P, Richir S (2021). A study of the effects of a natural virtual environment on creativity during a product design activity. Think Skills. Creat..

[45] Ulrich, R. S. Aesthetic and affective response to natural environment in *Behavior and the natural environment* (ed. Altman, I. & Wohlwill, J. F.) 85–125 (Plenum Press, 1983).

[46] Ulrich RS (1991). Stress recovery during exposure to natural and urban environments. J. Environ. Psychol..

[47] Bratman GN, Daily GC, Levy BJ, Gross JJ (2015). The benefits of nature experience: improved affect and cognition. Landsc. Urban Plan..

[48] DeCaro, M. S., Van Stockum, C. A., Jr & Wieth, M. B. When higher working memory capacity hinders insight. *J. Exp. Psychol. Learn. Mem. Cognit.* **42**, 39-49 (2016). 10.1037/xlm0000152 26120772

[49] Knaust, T. *et al*. Exposure to virtual nature: the impact of different immersion levels on skin conductance level, heart rate, and perceived relaxation. *Virtual. Real*. (2021).

[50] Faul F, Erdfelder E, Lang AG, Buchner A (2007). G*Power 3: a flexible statistical power analysis program for the social, behavioral, and biomedical sciences. Behav. Res. Methods..

[51] Braithwaite, J. J., Watson, D. G., Jones, R. & Rowe, M. *A guide for analysing electrodermal activity (EDA) & Skin Conductance Responses (SCRs) for psychological experiments* (University of Birmingham, 2013).

[52] Orita R, Hattori M, Nishida Y (2018). Development of a Japanese Remote Associates Task as insight problems. Jpn. J. Psychol..

[53] Watson D, Clark LA, Tellegen A (1988). Development and validation of brief measures of positive and negative affect: the PANAS scales. J. Pers. Soc. Psychol..

[54] Sato A, Yasuda A (2001). Development of the Japanese version of Positive and Negative Affect Schedule (PANAS) scales. Japanese J. Pers..

[55] Russell JA (1980). A circumplex model of affect. J. Pers. Soc. Psychol..

[56] Miyake, S. & Kumashiro, M. Subjective mental workload assessment technique-an introduction to NASA-TLX and SWAT and a proposal of simple scoring methods. *Jpn. J. Ergon.* **29**, 399–408. (in Japanese) (1993).

[57] Hart, S. G. & Staveland, L. E. Development of NASA-TLX (Task Load Index): results of empirical and theoretical research in *Human mental workload* (eds. Hancock, P. A. & Meshkati, N.) 139–183 (North-Holland Press, 1988).

[58] Merlo, L. J., Stone, A. M. & Bibbey, A. Measuring problematic mobile phone use: development and preliminary psychometric properties of the PUMP scale. *J. Addict.* 24826371 (2013). 10.1155/2013/912807 PMC4008508 24826371

[59] El-Sheikh M, Keller PS, Erath SA (2007). Marital conflict and risk for child maladjustment over time: skin conductance level reactivity as a vulnerability factor. J. Abnorm. Child Psychol..

[60] Clark, A. *Natural-born cyborgs: minds, technologies, and the future of human intelligence* (Oxford University Press, 2003).

[61] Brich, I.R., Bause, I.M., Hesse, F.W. & Wesslein, A.K. How spatial information structuring in an interactive technological environment affects decision performance under working memory load. *Comput. Hum. Behav*. **123** (2021).

[62] Liu WJ, Kitamura A, Sinohara K (2021). Characteristics of distraction caused by the presence of a smartphone in workplace. Jpn. J. Ergon..

[63] Tanil CT, Yong MH (2020). Mobile phones: the effect of its presence on learning and memory. PLoS ONE.

[64] Johannes N, Veling H, Verwijmeren T, Buijzen M (2019). Hard to resist? The effect of smartphone visibility and notifications on response inhibition. J. Media Psychol. Theor. Methods Appl..

[65] Crowley JP, Allred RJ, Follon J, Volkmer C (2018). Replication of the mere presence hypothesis: the effects of cell phones on face-to-face conversations. Commun. Stud..

[66] Hartmann M, Martarelli CS, Reber TP, Rothen N (2020). Does a smartphone on the desk drain our brain? No evidence of cognitive costs due to smartphone presence in a short-term and prospective memory task. Conscious. Cognit..

[67] Linares C, Sellier AL (2021). How bad is the mere presence of a phone? A replication of Przybylski and Weinstein (2013) and an extension to creativity. PLoS ONE.

[68] Bratman GN, Daily GC, Levy BJ, Gross JJ (2015). The benefits of nature experience: improved affect and cognition. Landsc Urban Plan.

[69] Dorfman L, Martindale C, Gassimova V, Vartanian O (2008). Creativity and speed of information processing: a double dissociation involving elementary versus inhibitory cognitive tasks. Pers. Individ. Differ..

[70] Mednick SA (1962). The associative basis of the creative process. Psychol. Rev..

